# The social representations of diagnosing Lyme disease

**DOI:** 10.1371/journal.pone.0276800

**Published:** 2023-02-09

**Authors:** C. Puppo, Y. Hansmann, L. Moinot, X. Duval, C. Chirouze, M. Préau

**Affiliations:** 1 UMR1296, Université Lyon 2, Lyon, France; 2 CHRU de Strasbourg, Strasbourg, France; 3 Univ. Bordeaux, ISPED, INSERM Bordeaux Population Health Research Center, UMR 1219, CIC1401-EC, CHU Bordeaux, Bordeaux, France; 4 Hôpital Bichat-Claude Bernard, Paris, France; 5 CHRU de Besançon, Besançon, France; Tufts University Cummings School of Veterinary Medicine, UNITED STATES

## Abstract

Social science studies on the controversy surrounding Lyme disease (LD) focused on the opposition between the “mainstream” and biomedical approach on one side and the “Lyme-literate” one on the other side, the latter claiming the existence of the chronic form of LD. The qualitative and exploratory study ‘C18-48 Quali-Explo-PIQTIQ’ (2019) investigated the social representations of LD in patients bitten by a tick. Twenty-four semi-structured interviews were conducted in three French medical units. Thematic and patient trajectory analyses were performed. Our results showed that, after the tick bite, some patients presented an “illness without disease” condition, characterised by uncertainty. In some cases, they consulted “Lyme-literate” health providers and received a diagnosis of chronic LD. This diagnosis was obtained by prescribing unassessed biological testing, providing an objective result and clinical categorisation. Unlike literature on the “Lyme-literate” approach, this diagnostic procedure involved some biomedical operations.

## Introduction

Lyme disease (LD) is a tick-borne bacterial infection and a topic of considerable ongoing scientific controversy not only in the USA but also in some European countries such as France. This controversy surrounds the symptomatology, diagnostic tools, treatment efficacy and whether or not LD is chronic [1].

Nowadays, scientific data about tick-borne disease representations and perception are very sparse [2]. Generally speaking, few studies have taken the psychosocial factors, experiences and representations of patients bitten by ticks into consideration. Some studies on LD controversy [3, 4] described it as the opposition between individuals supporting the “mainstream” or “orthodox” standard of care and those in favour of the so-called “Lyme-literate”or “heterodox” care and the inclusion of the chronic form of LD. The latter criticised the former on the following grounds in particular: 1) for failing to realise that the causative agent (i.e. *Borrelia burgdorferi)* persists after standard antibiotic therapy; 2) on the use of standard diagnostic tools (serological tests) deemed inaccurate by “Lyme-literate” individuals; 3) on the use of chronic therapy, since Lyme is conceived as a chronic disease.

More globally, in social science literature, this controversy has exemplified the opposition between a biomedical approach (represented by “mainstream” care) on the one hand [5], and a symptom-based approach, which is said to better embrace the ideals of health democracy (represented by “Lyme-literate” care) and patient-based care, on the other hand. Further investigation along these lines would show that the “mainstream” approach is linked to the assessment of LD as an objective disease, while the “Lyme-literate” approach views LD as a subjective illness. The “mainstream” and biomedical approach would opt for diagnosis on the basis of clinical categorisation, while the “Lyme-literate” approach would focus on a process involving different points of view in the investigation, interpretation and negotiation of treatment decisions regarding patient pain and/or patient health issues [6–8]. Actually, diagnosis can be conceived as both category and process [6, 7]. As category, diagnosis allows integrate the patient’s suffering in nosologic classification structures, separated by neat borders [9]. In this sense, diagnosing means stabilizing a phenomenon from a cognitive point of view, to treat it systematically. Instead, as process, diagnosis is a dynamic activity concerning investigation, decision, negotiation, based on clinical judgment as well as on biomedical technologies [10]. So, diagnosis as process, more than just a denomination, is the way of thinking the disease. It is a nonlinear process of attempts and mistakes [7], a complex and dynamic process, resulting from multiple perspectives and from emotional and relational factors [11].

The context of controversy highlights the structural and epistemological composing diagnosis [12], as well as the political and ethical issues [13]. Therefore, the concept of diagnosis allows to explore the borders between what is medical and what is social, and to investigate the way these borders are established.

Specifically to LD, Dumes (2019) [3] defined it as a “useful heuristic to think through biomedical explainability and its limits”, because of the frequent emergence of health problems that are clinically non-specific to LD and are not categorised as clinical labels. This condition is also experienced by patients living with medically unexplained symptoms (MUS), who often encounter uncertainty, delegitimisation, which deeply impacts their quality of life [14]. As outlined by Kirmayer *et al*. [15], in social science literature, some authors explain MUS through an interactional approach, arguing that the emergence thereof is associated with difficult negotiations between physicians and patients in terms of the meaning of the disease concept [16–18]. Others have explained MUS through a social approach and sociosomatic theories, arguing that illness can also result from social conflicts and can be embedded in the social and cultural context [19, 20].

However, in both cases, literature about MUS showed that patients living this condition feel to be stigmatized, both by healthcare providers and their family. The feeling to not be believed about one’s symptoms is accentuated by the subjectivity and invisibility of troubles. Obtaining a diagnosis for legitimating illness is needed not only for legal and insurance reasons [21], but also to give sense to owns’ experience [22]. Psychosomatic and psychological diagnosis or explanations imply for patients “that the illness is not as real as physical disease” [15, 23]; doctors can use them as “blame-shifting device” [23, 24], and MUS sufferers can feel their identity attacked [23]. Moreover, some studies showed that both doctors and patients feel distrust and hostility during consultation, not necessarily expressing it [17].

More specifically, Aronowitz [1] traced the history of the development of LD as a disease, heralding it as a biomedical success story. According to this author, diagnosis of chronic LD can be seen as a specific issue of a more general question in chronic disease: the distinction between disease and illness.

I have aimed to demonstrate how Lyme disease has been “constructed” or “negotiated” rather than discovered. […] By juxtaposing lay and medical attitudes and accounts of this recent phenomenon, we see how Lyme disease embodies and reflects aspects of our current and past beliefs about sickness, and how these beliefs, rather than being marginal influences on a fundamental biological reality, have shaped almost every aspect of medical practice and the lay response (pp. 107–108).

Based on these concepts (i.e. illness and disease), our hypothesis is that patients’ social representations valorize LD as illness rather than disease, in line with social science literature about the subject. Patients’ social representations of LD would be based on their symptoms’ experience, more than on biomedical tools and results.

The aim of the present article is not to review the controversial arguments and debates about LD: in a context of scientific and social controversy, we wonder how the diagnostic tools and the clinical interpretation of health problems are perceived in the patients’ experience. Given the aim of this article, a data focus and an empirical approach appear to be essential. Adopting qualitative methods to explore the subjective living of patients enabled us to thoroughly investigate the meaning given to this experience and to these unexplored social representations. The theory of social representations allowed us to understand how science permeates society and is shaped and re-worked via this process [25]. One of the qualitative methods’ aim is to seize the singularity and complexity of social phenomena [26], as well as the sense people give to their behaviors. Adopting qualitative methods makes it possible to develop a holistic and global approach and a comprehensive epistemology. As Flick [27] argues, “qualitative methods are used to discover how people deal with certain issues in everyday life or institutional practice”. To investigate social representations as social forms of knowledge, studying the “subjective meanings and mundane experience and practices” (22), for example through semi-directive interviews, seems to be very useful.

## Material and methods

Quali-Explo-PIQTIQ was a French psychosocial exploratory qualitative study, conducted in 2019, which enrolled patients bitten by a tick in 2017 and 2018 whether or not they subsequently developed a tick-borne disease (TBD). It aimed to capture the experiences and representations of these people regarding tick bites and LD. The study population was recruited from patients who either attended the Infectious Diseases Unit at Strasbourg Hospital or visited a general practitioner (GP) at the Health Clinic (*Maison de Santé*) in Schirmeck, both sites being located in the Grand Est (formerly Alsace) region of France, or who attended the Infectious Diseases Unit at the Besançon Hospital, located in the adjacent Bourgogne Franche-Comté region. Between June and September 2019, semi-structured and exploratory interviews were conducted by a social sciences researcher with 24 patients who were enrolled in the study by healthcare professionals at the three sites. Adult patients, having been bitten by a tick in the two years preceding our data collection, were included in the study. No other inclusion or exclusion criteria were defined: since our approach was holistic and comprehensive, every patient experience after the tick bite (being symptomatic without a diagnosis, being asymptomatic, having received a tick borne disease diagnosis) had been taken into account.

Each interview lasted approximately one hour and explored the following areas: participants’ experience of tick bites; their perception of the health risk associated with tick bites; their relationship with individual healthcare professionals and, more generally, with medical institutions; the emergence and management of health problems (only certain patients as others did not experience any problems but sought medical advice out of concern); the experience (for some) of a positive diagnosis for LD and of prescribed treatments; the support offered by loved ones; participant access to medical information about tick bites and LD; their coping strategies; their quality of life. The rationale for this exploratory qualitative as opposed to quantitative approach was that patients’ open-ended discourse would lead to the emergence of topics that we were unaware of or had not previously contemplated. Written informed consent prior to enrolment was mandatory. Anonymous personal identifiers were used for each participant. Interviewees were asked to complete a form collecting socio-demographic data at the beginning of the interview. Quali-Explo-PIQTIQ was authorised by the CEEI (*Comité d’Evaluation Ethique de l’Inserm*, *IRB 00003888*, *approval number 19–567*).

### Analysis

Both a thematic analysis and patient trajectory analysis were performed.

### Thematic analysis

The Computer Assisted Qualitative Data Analysis Software (CAQDAS) package ATLAS.ti [28] was used to apply the iterative and comprehensive process required for the thematic analysis of transcribed discourses. Primary discourse themes were identified as follows: characteristics of patients’ health problems; the influence of loved ones and of the media on the health problems’ attributions; patients’ relationship of trust/mistrust/negotiation with healthcare professionals; their reaction to the diagnosis of LD; their trust/mistrust of serological tests; their use of alternative medicine; their adoption/evolution/abandonment of prevention strategies to prevent tick bites.

### Patient trajectory analysis

Upon completion of the thematic analysis, we realised the value of implementing a temporal approach for the study. Consequently, we then conducted a patient trajectory analysis using participants’ discourses about their experience of their illness [29]. More specifically, the choice to conduct a patient trajectory analysis was driven by our desire to accentuate the temporal perspective. The concept of patient trajectory refers to the patient’s experience of their disease and the subjective process of suffering [29, 30]. In the present qualitative study, we used this concept to explore patients’ social representations and their evolutions. This approach helped us to investigate, from a temporal perspective, the various stages of patients’ experience with the disease itself, from the possible emergence of health problems to their reaction to the diagnosis, their adherence to medical prescriptions, etc. In literature, this approach was traditionally used to investigate representations of chronic diseases, but over time other diseases [31] with uncertain trajectories [32–34] also began to be explored, enabling researchers to investigate patients’ management of their subjective experience of the disease, while taking account of its evolution [35, 36]. In this sense, the subjective chronological flow does not necessarily correspond to the natural evolution of the disease. The patient trajectory approach reveals the disease’s social component in terms of disease onset, evolution and (possible) end.

We identified two main stages in patient trajectories: ‘pre-diagnostic work’ and ‘diagnostic work’ [37]. Pre-diagnostic work in our case involved the patient in trying to understand the cause of their clinical problems before their first clinical consultation. The diagnostic work stage commenced at the first consultation with a healthcare professional.

## Results

### The study population

Quali-Explo-PIQTIQ’s study sample comprised patients bitten by a tick between 2017 and 2018 in France who subsequently sought medical advice in one of the three afore-mentioned recruitment centres for a related reason, and who were assigned at the enrolment phase to one of the three clinical categories outlined above. Of the 33 individuals recruited, only 24 were subsequently interviewed. The nine patients excluded either no longer wished to participate or withdrew for practical reasons (for example, they had some personal problems during the period of the data collection or they could not be present at the interview for some logistic reasons). Interviewees were aged between 33 and 78 years old (median age = 57.5 years old). Their sociodemographic and clinical characteristics are presented in Table 1.

**Table 1:** Characteristics of the study population (n = 24).

***Median age***	57.5 years old
***Sex (n)***	
Men	10
Women	14
***Study Recruitment Site (n)***	
Besançon Hospital	7
Strasbourg Hospital	8
Maison de Santé in Schirmeck	9
***Clinical condition (n)***	
Asymptomatic patients	5
Symptomatic patients not diagnosed with Lyme disease (various symptoms persisting for at least 6 months)	5
Patients diagnosed with Lyme disease:	14
Patients diagnosed by an infectious disease specialist at Strasbourg Hospital or Besançon Hospital	4
Patients diagnosed by other health care providers (dermatologists, rheumatologists) with subsequent confirmation of diagnosis by an infectious disease specialist at Strasbourg Hospital or Besançon Hospital	3
Patients diagnosed by a GP at the *Maison de Santé* (Schirmeck)	3
Patients diagnosed by a GP (chronic Lyme diagnosis)	4
***Educational level (%)***	71% ≥ high school diploma
***Socio-professional situation (%)***	
Employed	44
Retired	42
Low-income benefit, disability benefit	8
Housewife/house husband	4
Self-employed	2
***Self-perceived financial situation (%)***	
Comfortable	42
Getting by	37.5
Just making ends meet	12.5
Difficult to make ends meet	8
***Perception of current living environment (%)***	
Positive	42
Quite positive	50
Quite negative	8

In this article, we will focus on the results concerning the ‘diagnostic work’, and, more specifically, on the patients’ social representations of the clinical diagnosis and its tools (Table 2). This stage of the patient trajectory showed how patients perceived the controversy surrounding LD through its diagnosis, as well as the way in which science is integrated in their social representations and adapted to their common sense communication.

**Table 2:** Study’s main results.

**The experience of uncertainty**	The difficulty in interpreting health problems after a tick bite
	The feeling of incongruence between the presence of subjective health problems and the absence of clinically objective results
	The impact of unexplained health problems on the patients’ quality of life
**Interpreting persistent health problems: the chronic Lyme diagnosis**	Consulting “Lyme- literate” physicians when no explanation for persistent health problems could be found
	Consulting “Lyme-literate” physicians after a previous LD diagnosis: opposing “mainstream” care
	Consulting “Lyme-literate” physicians after a previous LD diagnosis: complementing “mainstream” care
**The representations of Chronic Lyme disease diagnosis**	The merits of the “Lyme- literate” biological tests
	Devaluation of the clinical interpretation portrayed by “mainstream” care
	Adopting biomedical concepts
	Checking your own health status through “Lyme-literate” biological testing

In this article, we will adopt the terms presented above (i.e. “mainstream” and “Lyme literature”) for two different reasons:

- We found this distinction and these (or similar) terms in the literature [3, 4] to refer to the two sides of the LD controversy. They will be useful from an analytical perspective. Nevertheless, as Dumes (2019) also reported, the so-called “Lyme-literature” physicians have mostly been biomedically trained (with some incorporating alternative medicine in their practice) and do not have specific training in LD.
- We found this distinction and these denominations in our field. In actual fact, the controversy is evident in patients’ social representations of LD. In our study, some participants reported that they consulted “Lyme doctors”: these were mainly GPs (sometimes excluded from the official French Medical Association), but also osteopaths, and homeopathic physicians. In general, our participants used the term “Lyme doctors” to refer to physicians who recognise the existence of chronic LD and prescribe unassessed biological tests and treatments other than those prescribed by “mainstream” physicians. Consequently, “Lyme doctors” in our field can be identified as “Lyme-literate physicians” in the literature.

### The experience of uncertainty

After a tick bite, participants reported various health problems, some of which were not clinically specific to LD. For the purpose of our analysis and from a psychosocial perspective, it was possible to differentiate between health problems reported by our participants after the tick bite as follows: 1) visible or measurable, “clinically objective” signs, such as erythema migrans, fever, joint swelling, facial paralysis, vomiting, etc.; 2) invisible, “subjective” health problems, such as fatigue, nausea, memory problems, poor concentration and lack of organisation, sensation of stiff joints, sensation of heat or burning, diffuse pain, etc. [3].

We will focus here on invisible and subjective health problems.

After the tick bite and the possible emergence of these health problems, some patients underwent some medical examinations (i.e. MRI scan, X-rays, ultrasound scans, etc.) to understand the reason for their pain. The often negative results of these medical examinations provoked a feeling of incongruence between the presence of subjective health problems and the absence of clinically objective results in our patients. These clinically unexplained health problems impacted the patients’ quality of life (in physical, psychological and social terms), and sometimes provoked stigma experiences [1]. Because of their invisibility, these health problems were actually more complicated to report and explain to physicians compared to visible and clinically objective signs. At times, patients also found it difficult to share their experience with their loved ones. The seriousness of health-related problems was subjectively valued by patients through the difficulty experienced in carrying out daily living activities, which were not specifically meaningful from a clinical perspective but were important from the patient standpoint.

These results are condensed in Table 3.

**Table 3:** The experience of uncertainty.

**The difficulty in interpreting health problems after a tick bite**	*I mean uh*, *uh*, *Lyme disease symptoms*, *they say it attacks the joints uh*, *and I’ve got a lot of*, *of pain in my joints*, *but I can put that down to work too*, *so it’s complicated*, *I find it hard to*, *hard to make a distinction*, *so I don’t know if it comes from that or if it’s the work that’s physical*, *and so it comes from*, *from working* *(34-year-old woman)*.
**The feeling of incongruence between the presence of subjective health problems and the absence of clinically objective results**	*So*, *you ask yourself the question… I had an MRI of the brain*, *they found nothing*, *and*, *well*, *I began to look for explanations*, *because no one today can still explain to me what was happening to me*, *so I*, *I didn’t understand*. *They looked for multiple sclerosis*, *they looked for other*, *other possibilities*, *but since everything was negative*, *I still say to myself*, *there is an explanation*. *(49-year-old woman)* *They looked for multiple sclerosis*, *they looked for other*, *other possibilities*, *but since everything was negative*, *I still say to myself*, *there is an explanation*. *(49-year-old woman)*
**The impact of unexplained health problems on the patients’ quality of life**	*What’s difficult*, *it’s just how uh… they don’t do everything together; they do a test*, *uh… I wait three weeks*, *four weeks for an appointment*, *then*, *I wait again for the rheumatologist*, *and then they proceed step by step*, *which means that it’s very*, *very*, *long uh… until you can get rid of it*. *As you go along uh… […] Next week*, *I still have to do a uh…*, *blood test to look for*, *uh…*, *and uh… another genetic disease that they haven’t tested for yet*, *but I still don’t know uh…*, *and what’s more*, *it’s getting worse* *(53-year-old woman)*. *My*, *my aunt*, *she is*, *me*, *I remember*, *it’s my aunt*, *she adores me and she really is a very nice person*, *who is very*.* *.* *.*who is good at her job and also has a scientific approach*, *who is very interested in that*, *she should have retired by now but she hasn’t retired yet because she*, *because she is passionate about that*, *her passion and then medicine*, *well…she is*, *it was clear to her when I told her the symptoms*, *it’s cruralgia [Cruralgia can be defined as the anterior pain in the thigh or shin]*, *I had that*, *so on*, *so forth*, *when it started to develop a bit*, *but it was almost as if she didn’t get a bit angry when she told me you were talking rubbish to me*. *It’s not possible*, *because as she had detected that it was cruralgia*, *well cruralgia cannot develop as it was developing*, *except that I’m sorry*, *yes*, *that’s how it was*, *it was like that* *(42-year-old man)*.

### Interpretation of persistent health problems: The chronic Lyme disease diagnosis

As discussed above, when subjective health problems persisted, most patients experienced a period of uncertainty about their health status and the label used to identify their health problems [38]. Our results showed that these health problems were sometimes attributed to LD by patients and their loved ones and/or by physicians. In the last case, LD was clinically diagnosed by the GP (n = 7) and/or by “mainstream” physicians (n = 7) (in most cases infectious disease specialists and rheumatologists) and/or by “Lyme-literate” health-care providers (n = 5). In the last case (i.e. when the LD diagnosis was presented by a “Lyme-literate” health-care provider), it was a case of chronic LD.

“Lyme-literate” physicians were consulted by some patients experiencing uncertainty for two main reasons.

1. They were consulted when the GP and/or “mainstream” care specialists had not categorised the patients’ health problems as LD symptoms, because of their clinical non-specificity. In the absence of any other diagnosis, the patients felt that their condition had not been recognised for two main reasons. Firstly, being aware of the controversy surrounding LD, they believed that the medical and scientific research on LD was incomplete and potentially false. Secondly, they had come up with a self-diagnosis based on the loved ones’ advice, some medical or paramedical opinion and social comparison with people reporting LD symptoms.
2. “Lyme-literate” physicians were consulted following LD diagnosis made by the official and “mainstream” care providers, and after having been treated for this with standard antibiotic therapy. In this case, the persistence of some subjective health problems made the patient look for other medical opinions (i.e. the opinions of the “Lyme-literate” physicians). According to “mainstream” care providers, persistent health problems are sequelae of LD, probably meant to disappear or decrease over time. Nevertheless, this explanation was not accepted by some patients, who decided to refer to “Lyme-literate” health-care providers. This choice was sometimes made to oppose “mainstream” care (2a) and, at other times, to complement it by seeking an alternative treatment rather than complete follow-up (2b). In case 2a), patients often received a double LD diagnosis, one of which (i.e. the one delivered by the “Lyme literate” health-care provider) was chronic.

These results are resumed in Table 4.

**Table 4:** Interpreting persistent health problems: The chronic Lyme disease diagnosis.

**1. Consulting “Lyme-literate” physicians when no explanation for persistent health problems could be found**	*Uh*.* *.* *. *especially in relation to the*, *um*, *ultrasound scans s and X-rays that I had done*, *which had shown nothing*, *absolutely nothing*, *and then*.* *.* *., *well afterwards*, *there was*.* *.* *. *no solution either*, *and so I had heard about*.* *.* *., *from the department*, *Professor X*. *I made an appointment*, *and then he redid my*, *well*, *finally I had my blood tests redone*, *which showed the same*.* *.* *. *the same things*, *but*, *according to him*, *the symptoms I have do not match what we currently know about Lyme disease*. *[*.* *.* *.*] well that’s what you hear everywhere*, *that in fact*.* *.* *.*very few people are diagnosed with*.* *.* *.*Lyme disease*. *(53-year-old woman)*
**2a. Consulting “Lyme- literate” physicians after a previous LD diagnosis: opposing “mainstream” care**	*So I was seen several months later by the infectious diseases specialist*.* *.* *. *who said to me "but*, *you’ve had the course of antibiotics*, *you’re cured"*. *"You’re cured"*. *Well*, *anyway*, *frankly*, *I think they treat people like idiots*. *[…] He didn’t say*, *well*, *"It’s in your head" but I (cough)*, *that’s what he meant*, *like well*, *if you’ve other symptoms*, *there’s*, *it’s…The fact I had the procedure*, *and had taken a course of antibiotics for twenty-one days*, *that was*, *that was*, *like*.* *.* *.*That had to be done*, *to resolve the issue*, *so that it would be behind me*, *and I that I could turn the page*. *[…] I don’t feel cured but I’m well on the way*. *I’d say on the right track*. *But not cured*. *But I’ll fight it (Laughs)*. *(55-year-old woman)*
**2b. Consulting “Lyme- literate” physicians after a previous LD diagnosis: complementing “mainstream” care**	*Essential oils*, *yes but*.* *.* *. *generally no [I don’t take any] but I have taken this product for almost 18 months*. *It’s horrible*, *but I took it*, *uh*.* *.* *. *when I became interested in Lyme*, *there’s Tic Tox* [*Tic-Tox is an essential oils therapy*, *which use has been banned in France in 2012*, *due to neuro-toxicity risks associated with this treatment]*, *which was the product that caused a scandal*, *which is banned in France*, *and I said to myself*, *how can I get this product*, *which is*, *which is not normal*, *so you try it out yourself*, *you look for the medicinal product or the product yourself*. *But then I came across something on the Internet that said it’s the equivalent of Tic Soc*, *Tic Tox*, *except that it’s available over the counter*. *I told professor X ["mainstream" physician]*, *well… he told me "medically*, *it’s worth it*, *I can’t tell you anything because it’s not medical*, *there’s no*.* *.* *. *no analysis to prove that it’s good*, *if it does you good*, *and you don’t get any side effects*, *continue to take it”*. *So I have these essential oil products*, *from*, *I don’t know*, *where it says on it*, *it boosts the immune system against the flu*, *whatever*, *I take 20 drops every day*, *and I’ve been doing that for 18 months*. *(54-year-old man)*

### The representations of chronic Lyme disease diagnosis

In a psychosocial setting, consulting “Lyme-literate” physicians allowed participants to “become” chronic LD patients, sometimes after having previously had a “mainstream” LD diagnosis [see (2) in the paragraph above]. Study participants reported that chronic LD diagnosis was feasible through the prescription of so-called unassessed biological tests in particular. These tests, the efficacy of which is debated in the controversy surrounding LD, are not acknowledged by the official French health system or “mainstream” approach (20) [39], and were often performed in a German private clinic. They were prescribed by “Lyme-literate” health-care providers and described by most patients as more precise, reliable and scientific than those proposed through the “mainstream” care system. Patients reported that the former were more detailed and informative about the organs affected by chronic LD as well as about concomitant infections triggered by tick bites. Consequently, these patients felt reassured by undergoing regular tests. Patients perceived these regular checks as one way of controlling their chronic LD and its clinical course, and highlighting the fact that “mainstream” serological testing had not identified [see (1) above] or no longer identified [see (2) above] the active LD bacterium (i.e. *Borrelia burgdorferi*) because it was less sophisticated. Based on unassessed biological testing, the “Lyme-literate” physicians’ diagnosis portrayed chronic LD as a silent, dormant, persistent bacterium (these words were often used by participants during the interviews). Since, in this social representation, the Lyme bacterium is constantly in the body and could resurface at any time, these participants needed verification through regular checks. At the same time, the immunity system had to be boosted through prolonged intravenous antibiotic therapies and/or alternative medicines. The representation of LD as an active and dormant bacterium was shared by many participants and not only those diagnosed with chronic LD. These results are resumed in Table 5.

**Table 5:** The representations of chronic Lyme disease diagnosis.

**The perceived “merits” of the “Lyme-literate” biological tests**	*The last analysis was done here [medical unit proposing the "mainstream" care]*, *and I’m not happy*. *I would like to have had a bit more*, *information*, *in a bit more detail since it has come to light that borrelia affects the nervous system*, *borrelia affects the muscles*, *borrelia that*.* *.* *. *There you go*. *And there*, *that’s it*, *they have put everything in the same package*. *When you compare the blood test done here and the blood test done at X [medical unit offering the "Lyme-literate" tests]*, *you can’t believe it*. *You get the impression that it’s a rush job*. *The "Lyme-literate" physician] explained everything to me in great detail*..* *.* *.*] So they found that I had borrelia in my blood and then well*.* *.* *. *The blood test was really very*, *very well done*.* *.* *. *There were two pages of it*, *with different kinds of borrelia*. *[*.* *.* *.*] But when you are left in the dark like that*, *I can’t do that*. *I need to be told*,.* *.* *. *It’s like this*, *etc*. *Well*, *perhaps in the end it comes down to the details*. *I’m more of a scientist after all*. *(**70-year-old woman**)*. *I now know that I have Lyme disease because I had a repeat serology test*. *I had another serology test–more complex than what we do here*. *[*.* *.* *.*] Besides*, *nobody wanted to prescribe this serology test for me*, *so I had to pay for it with my own money*, *it cost me eighty euros to have this serology test*. *And now I know exactly which one*, *which bacteria I am infected with*, *well*, *I have four*, *I’m infected with four bacteria*..* *.* *.*] I know that one attacks the heart*, *one attacks the brain*, *one attacks the skin and one attacks the joints*. *Here [medical unit proposing the "mainstream" care] it was*, *it was*, *I was positive but uh without knowing which bacteria were involved*, *there we are*. *[*.* *.* *.*] My brother saw Dr X ["Lyme-literate" physician]afterwards and his daughter wanted to go with him*. *And Doctor X*, *when he saw his daughter*, *well I don’t know what*, *well what going on*, *he said "oh*, *you have to have the serology test"*. *And she did the test and she’s um and it’s positive*, *yes*, *it’s positive*. *(**55-year-old woman**)* *Well*, *she did something quite precise*, *ah*, *yes*, *and then she sent me to do an analysis in a much more precise laboratory for microbio*.* *.* *. *microbiotics*. *[*.* *.* *.*] It was really also to see how my body reacted*.* *.* *., *it was really extremely precise in relation to the types of Borreliosis (**33-year-old woman**)*.
**Devaluation of the clinical interpretation portrayed by “mainstream” care**	*He [the "mainstream" physician] listened to me and*, *well*, *that’s all*. *I didn’t see what he wrote down*, *but there was no examination*, *no treatment*, *there was nothing (**54-year-old man**)*. *On the other hand*, *he didn’t prescribe any tests for me*. *So*, *I didn’t have that much confidence in him*, *because I said to myself*, *well*, *he’s telling me that and he’s not doing any tests*, *he’s not asking me for tests*, *not even straightaway*, *but he’s prescribing these antibiotics for me and isn’t interested in finding out what the situation is going to be two months down the line (**33-year-old woman**)*. *But on the other hand*,.* *.* *. *so he told me that*, *without any additional blood tests or anything*. *[*.* *.* *.*] So that’s why*, *I*, *there you go*, *it’s hard to say*. *(**38-year-old man**)*. *It’s the one from 2009 ["mainstream" physician] who was starting to get on my nerves saying you don’t have Lyme disease*. *Yes*. *See what I mean*? *But I had some results nevertheless (**76-year-old woman**)*.
**Adopting biomedical concepts**	*If you’re tired*, *if you’re depressed*, *if you’re*, *well*, *the bacteria will take over*, *the disease will take over and then it could attack something or other*. *But that’s the fundamental principle*, *you have to*.* *.* *. *live with it*, *basically*. *[*.* *.* *.*] What worried me was that*.* *.* *. *well*, *it can*,.* *.* *. *it can*, *it can come back in a form*, *in another form*. *When you have the bacteria*, *you say to yourself*, *uh*.* *.* *.*"well*, *brace yourself–it’s going to attack" (**60-year-old woman**)*. *This Lyme disease*, *is hidden in the body and it’s as if*, *how you say*, *the bacteria*, *they form cysts and then they hide themselves and at the moment you still don’t have the option to detect it and what I have been told is true*, *that’s what I think (**53-year-old woman**)*. *So I understood because I tried to find out*, *to understand what was going on*, *so I understood that in fact*, *with antibiotic therapy*, *… it kills the bacteria*, *it attacks the bacteria and as a result it poisons the*, *the body*, *well it loads the body with toxins and as a result um*, *that’s it*, *the*, *the*, *the I think that the heart*, *the liver are really put to the test and*.* *.* *. *and this causes considerable fatigue*. *(**55-year-old woman**)*. *‘Cured’ that’s a word*, *eh*.* *.* *. *let’s say I*, *I*, *I can eh*.* *.* *. *actually*, *that’s where*, *where I don’t really know*. *I*, *I’m cured*, *in the sense that the bacteria uh*.* *.* *. *uh*.* *.* *. *borrelia uh*.* *.* *. *are uh*.* *.* *. *all dead*.* *.* *. *uh*.* *.* *. *and have disappeared from my body*.* *.* *. *uh eh*.* *.* *. *I’ve no idea (Noise) (Laughs)*. *Presumably uh*.* *.* *. *according to eh*.* *.* *. *the information that I have*, *the exchanges I’ve had with Professor X*, *the answer would be*, *yes*. *Uh*.* *.* *. *now I still have symptoms*. *So is it eh*.* *.* *. *due*, *due to a*, *a*, *a residual presence of*, *of bacteria uh*.* *.* *. *which*, *which*, *which is still lying around somewhere*.* *.* *. *In my body’s ‘inner windings’*, *I don’t know*. *(38 years old man)*.
**Checking your own health status through “Lyme-literate” biological testing**	*I had a serology test not long ago (noise) in October I think*, *[*.* *.* *.*] it’s always*, *it’s always positive*.* *.* *.*the*, *the search for*, *for antibodies*.* *.* *.*there you go…*, *the antibodies*, *it’s always positive*. *So*,.* *.* *. *it’s the same thing*, *they [the mainstream physicians] tell us*,.* *.* *. *"The antibodies will always be there*, *it will always be positive*, *you can do as many tests as you like*, *it will always be positive*.* The doctor I saw*, *a specialist in Lyme disease*, *didn’t give me any assurance -*, *she said "you’ll always have it [*.* *.* *.*] your antibodies*, *well there you go*, *you react to the disease*, *you have it*.* *.* *.*" (**60-year-old woman**)* *I feel good*, *when I take blood samples*,.* *.* *. *I take blood samples that are suitable*, *I base my results on the blood samples*. *So yes maybe I would do another blood test*. *[*.* *.* *.*] To see whether or not it has changed (**66-year-old man**)*. *Once a year*, *in fact*, *we used to do a blood test for Lyme disease*.* *.* *. *to know where I was but it was just a check*. *So after the 2017 injection*, *we did it more regularly because we saw that there was a reaction*, *there were figures*. *So as a result my doctor was rather concerned*, *so we did tests a bit more often*. *That’s it*. *[*.* *.* *.*] But since I had Dr X ("mainstream" physician) I stopped doing them because he told me that I didn’t have Lyme disease*. *I’ll do another one anyway to monitor*, *but I’m not going to*, *I’ll do one a year to see (**60-year-old woman**)*

## Discussion

Other TBD (such as babesiosis, ehrlichiosis, Rocky Mountain Spotted Fever, anaplasmosis, etc.) are not concerned by scientific and social controversies and media coverage as Lyme. However, the Lyme-literate approach draws the attention on them as coinfections and occulted infections, which transmission could provoke serious symptoms. The mainstream approach, on the other side, affirms that these statements are not supported by validated biomedical evidences.

However, social sciences on TBD especially focused on prevention strategies and on their acceptance. In general, literature on vector borne diseases–not necessarily transmitted by ticks–investigate the interspecies relations, through a One Health approach, not only from a biological perspective but also from a social one [40].

In this article, we explored patients’ representations of “diagnostic work” following a tick bite, in a context of scientific and social controversy.

Firstly, we described the patients’ experience of uncertainty due to subjective and persistent health problems. This uncertainty can affect various aspects of the patients’ quality of life, especially since patients felt that they did not match any clinical label. When study participants were uncertain about their health problems, they experienced an ‘illness without disease’ condition [41]. In other words, they experienced some subjective health problems but they could not attach any clinical labels. In this sense, illness occurred in the absence of a clinically proven disease. Nevertheless, this condition is not specific to LD, since it is also quite common in the context of other diseases, such as fibromyalgia [42], chronic diseases [43] and especially MUS. These conditions have in common “the especially problematic negotiations between doctor and patient concerning what is disease and what is illness” [1].

Secondly, we pointed out the reasons why patients consulted health-care providers supporting the “Lyme-literate” approach rather than advocates of the “mainstream” approach, tending to adopt a more or less counter approach towards the latter.

Finally, we reported how patients described unassessed biological testing prescribed by “Lyme-literate” health-care providers, leading to the diagnosis of chronic LD. These tests, which are not acknowledged by the French health-care system, were perceived by patients to be more precise, scientific and reliable compared to the official care approach. As far as the patients are concerned, serological testing proposed by “Lyme-literate” health-care providers gives a name to their condition and explains persistent health problems as symptoms of an active disease (i.e. chronic LD) rather than as the sequelae of previous, cured LD or MUS.

Specifies about the French context can be pointed out.

First, our study mostly took place at the border between France and Germany. Patients we interviewed often went to Germany to be diagnosed (and treated) with LD, through non-validated tests and alternative treatments. More specifically, care structuration differs between France and Germany. In Germany, “heilpraktiker” are healthcare providers—not necessarily doctors—who practice alternative medicine and sometimes have opened their diagnostic lab. In France, this kind of care does not exist: this is why the “heilpraktiker” can represent a concurrence for French “mainstream” doctors. The German practices were often evoked by the population of our study, living at the border between France and Germany. Therefore, the French LD diagnosis representations were shaped through the comparison with the German ones. Furthermore, the media coverage about LD controversy in France has been boosted by the publication of the “Plan national de lutte contre la maladie de Lyme” (National Plan against Lyme disease) [44], launcher by the French Health Minister in 2016 [45].

We will now present some epistemological issues arising from the analysis of the ‘diagnostic work’ as perceived and discussed by study participants.

As mentioned in the introduction, according to the literature, the “Lyme-literate” approach is opposed to the “mainstream”, biomedical approach, which is accused of being too focused on the technical tools used to measure the disease rather than on the patient’s experience of the illness. Nevertheless, and paradoxically, our results showed that–at least in terms of patient perception–diagnostic testing was almost systematically used and highlighted by “Lyme-literate” health care providers as objective, reliable technical tools for measuring and checking chronic LD. This illustration is part of medicalisation involving the lay world [46], but is also made “by the lay world” [47], because it is integrated in patients’ social representations.

During the ‘diagnostic work’, some patients assigned the “power” to the biological objective results to disprove the clinical interpretation. More specifically, the biological objective results were generated by the unassessed biological testing proposed by the “Lyme-literate” approach; the clinical interpretation was preferred by the “mainstream” physicians. In actual fact, the latter interpreted the persistent health problems as LD sequelae or as other non-LD-related health problems via a clinical review of the patients’ health problems, warranting, in some cases, confirmation through serological testing. Thus, the clinical interpretation, based on the analysis of patients’ health problems by physicians supporting the “mainstream” care approach, was negatively perceived by patients, because it was seen as too subjective, partial and uncertain. On the contrary, the unassessed biological testing proposed by the “Lyme-literate” care pathway were presented by patients as real, objective evidence of their LD, and was sometimes considered to be more reliable and accurate compared to the same subjective symptoms they experienced.

Our results showed that patients consulting “Lyme-literate” physicians did not report on specific discussions about their health problems with these health-care providers. In portraying “Lyme-literate” care and diagnosis, these patients adopted some biomedical concepts and adapted them to common sense communication [17]. For example, the chronic LD bacterium–frequently mentioned by patients during the interviews—was represented as a constantly present, dormant object. In patients’ representations, this bacterium was an invisible and insidious object revealed as an objective sign through the “Lyme-literate” tests. These results corroborated what Soncco [48] argued about the “microbial agency” assigned to Borrelia by patients, especially to describe the bacterial persistence. As Augé and Herzlich [49] pointed out, the clinical approach influences not only patients’ representations of diseases but also of their bodies, as “fragmented” in symptoms, organs and functions.

In patients’ perceptions, the results of the “Lyme-literate” unassessed biological tests allowed subjective health problems [50] to become objective. Consequently, when health problems changed or regressed, these tests were used to determine the patients’ health as a matter of priority rather than the same health problems.

Social science literature on LD combined the “mainstream” approach with highlighting the objective disease and tools on the one hand, and the “Lyme-literate” approach with the focus on subjective illness and experience on the other hand. These dichotomies, when applied to other conditions, led to an important critical analysis of the biomedical approach and its use of technical-scientific innovations [51–53]. However, in the LD context, our result showed that this dichotomy should be explored in greater depth.

It is true to say that, from a psychosocial perspective, the “Lyme-literate” diagnosis was perceived by some participants as a way of dispelling uncertainty by giving a name to their condition [54]–a name that, in some cases, coincided with the self and lay diagnosis previously made by patients [55]. Nevertheless, our results showed that, when we conducted our interviews, the dispelling of uncertainty from a psychosocial perspective did not necessarily coincide with finding an effective treatment or disappearance of the persistent health problems, from a clinical perspective. However, as Dumes [56] argued, evidence based medicine (EBM) is a way to “bio-legitimate” and normalize ones’ health problems.

Nevertheless, contrary to our hypothesis, uncertainty was brought to a close by some biomedical strategies—at least as far as the patients were concerned:

- Considering diagnosis as a categorisation tool (i.e. health-care providers supporting the “Lyme-literate” approach assigning a clinical label to the patients’ condition) as opposed to a process, the latter being privileged through the clinical interpretation of patients’ health problems and a biopsychosocial approach. This categorisation performed by the “Lyme-literate” approach–by labelling health problems and onset of disease [57]–would help to increase the medicalization of LD.
- The use of unassessed biological testing to detect chronic LD–tests not acknowledged by the French healthcare system but emphasised by patients as objective tools to translate their subjective health problems into objective clinical signs. This strategy promotes the “alienation” [41] of the illness becoming a disease, and is linked to the uncertainty experienced by patients: “the greater the uncertainty, the more important it is to find a bodily trace […] and then make it tangible” [47].

From a methodological perspective, we retrospectively traced the patients’ trajectory from the time of the tick bite to investigate whether the said trajectory is influenced by psychosocial and clinical factors. The patient trajectory analysis carried out allowed us to critically analyze the three clinical categories assigned by healthcare professionals during the initial consultation (i.e. asymptomatic, symptomatic and affected by LD), in order to evaluate the application of a more dynamic and process-based approach. The comprehensive, holistic approach we adopted in this study allowed us to question at depth the dichotomy between a condition intended as an illness or as a disease.

In conclusion, in the case of LD, the “Lyme-literate” approach as opposed to biomedical care is not necessarily a less biomedical approach as far as patients’ perception. The representation of LD made by patients having consulted health-care providers supporting the “Lyme-literate” approach identified this condition as a disease more than an illness. Actually, patients demanded for and contributed to categorize their symptoms into a biomedical disease, to be measured and objectified through positive serological tests and the presence of an active bacterium in their bodies. Paradoxically, the portrayal of LD as an objective and measurable clinical object is actually more in keeping with the patients’ social representations of “Lyme-literate” care compared to the “mainstream” approach. Exploring the patients’ social representations of LD, and especially the way biomedical diagnosis and tools are interpreted, allows prevent “medical equivocations” [48] and facilitate the communication between patients and doctors’ narratives.
